# Neural mechanisms underlying visual pareidolia processing: An fMRI study

**DOI:** 10.12669/pjms.346.16140

**Published:** 2018

**Authors:** Gulsum Akdeniz, Sila Toker, Ibrahim Atli

**Affiliations:** 1 *Dr. Gulsum Akdeniz Assistant Professor Ankara Yildirim Beyazit University, Medicine Faculty, Electroneurophysiology Lab, and Yenimahalle Training and Research Hospital, Ankara, Turkey*; 2 *Sila Toker Clinical Psychologist MSc foundations of Clinical Psychology, Department of Psychology, Bournemouth University, Talbot Campus, Bournemouth, Dorset, United Kingdom*; 3 *Ibrahim Atli, PhD. Ankara Yildirim Beyazit University, Engineering and Natural Sciences, Ankara, Turkey*

**Keywords:** Face processing, fMRI, Fusiform face area, Pareidolia, Prefrontal cortex

## Abstract

**Objectives:**

Pareidolia is the interpretation of previously unseen and unrelated objects as familiar due to previous learning. The present study aimed to determine the specific brain areas that exhibited activation during real-face and face-pareidolia processing.

**Methods:**

Functional Magnetic Resonance Imaging (fMRI) scans were performed on 20 healthy subjects under real-face and face-pareidolia conditions in National Magnetic Resonance Research Center (UMRAM), Ankara, Turkey from April 2016 to January 2017. FSL software was used to conduct a FEAT higher level (group) analysis to identify the brain areas activated during real-face and face-pareidolia processing.

**Results:**

Under both the real-face and face-pareidolia conditions, activation was observed in the Prefrontal Cortex (PFCX), occipital cortex V1, occipital cortex V2, and inferior temporal regions. Also under both conditions, the same degree of activation was observed in the right Fusiform Face Area (FFA) and the right PFCX. On the other hand, PFCX activation was not evident under the real-face versus face scrambled or face-pareidolia versus pareidolia scrambled conditions.

**Conclusions:**

The present findings suggest that, as in real-face perception, face-pareidolia requires interaction between top-down and bottom-up brain regions including the FFA and frontal and occipitotemporal areas. Additionally, whole-brain analyses revealed that the right PFCX played an important role in processing real faces and in face pareidolia (illusory face perception), as did the FFA.

## INTRODUCTION

Of the visual stimuli that have a survival value, faces are among the most important because humans are familiar with perceiving them. Kanwisher et al.^1^ found that face stimuli evoked activation on the lateral side of the mid-fusiform gyrus, also called the Fusiform Face Area (FFA), and a Functional Magnetic Resonance Imaging (fMRI) study by Kanwisher and Yovel^2^ showed that the fusiform regions exhibited greater activation to faces than to letter strings or textures. Recent studies investigating the FFA have shown that this region exhibits significant activation for real faces as well as during face pareidolia.^3^-^5^ Face pareidolia refers to the attribution of real face traits to non-face objects due to illusory perceptions. This phenomenon typically occurs when non-face stimuli erroneously activate a connection between visual input areas and internal representations.^6^,^7^

Liu et al.^4^ investigated the neural and behavioural correlates of face pareidolia and showed that the FFA played a crucial role in the perception of real faces as well as the processing of illusory face perceptions. These authors also reported that the FFA showed higher activation during face pareidolia than during letter pareidolia. The neural mechanisms underlying face-pareidolia perception were proposed to include an interaction between bottom-up processing and top-down processing,^4^ and accordingly, the brain analyses in this study revealed that both frontal and occipitotemporal regions were important for processing face pareidolia. Occipitotemporal regions, including the primary visual cortex, enable the perception of visual input and are highly associated with bottom-up processing, whereas the frontal cortex is responsible for reasoning and is highly associated with top-down processing. When presented with illusory face stimuli that have components similar to normal faces, such as eyes and mouths, humans engage in top-down processing that creates associations with previous knowledge about real faces, which results in interpreting an illusory face as a real face.

Summerfield et al.^8^ reported that when a house was perceived as a face, the FFA exhibited greater activation and that the medial frontal region, which is responsible for decision making, also showed a high level of activation. These findings indicate that interactions between bottom-up and top-down modulations play an important role in face pareidolia. However, few studies^8^-^10^ have investigated the different and shared properties of the neural mechanisms underlying real-face and face-pareidolia processing. Thus, the present study investigated the neural mechanisms of actual face perception and face pareidolia to determine the relationship between face pareidolia and FFA activation.

## METHODS

The present study recruited 20 healthy right handed volunteers (14 women, 6 men, mean age: 26.50 ± 2.15 years). All subjects had normal or corrected to normal vision acuity and had no history of sensory system related pathology or neuro-psychiatric disorder. Two subjects were excluded from the study due to data transformation issues; thus, fMRI data from 18 individuals were collected and analysed. Individuals with claustrophobia were not selected for the experiment. Approval for the study was obtained from Ankara Yildirim Beyazit University, Medical Faculty Ethics Committee.

### Stimuli & Apparatus

**Fig.1:**
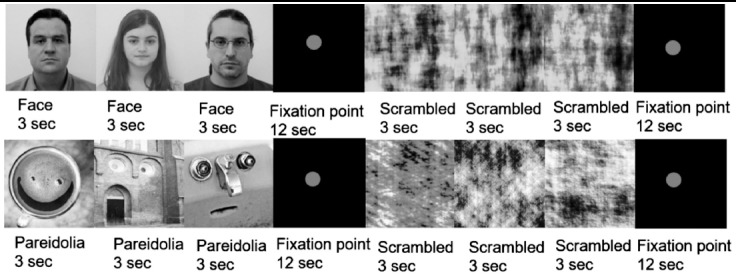
This block represents the real-face condition (upper row) and the face-pareidolia condition (lower row).

This research was conducted using fMRI to measure variations in the blood-oxygen-level-dependent (BOLD) activity of a group of neurons in a local population. For this study, 15 face photos were selected from among photos of 200 people obtained from the image processing laboratory of Centro Universitario da FEI. Face-pareidolia images were selected from Google Images by searching for “face pareidolia.” The real-face and face-pareidolia images were converted to grey scale to make their luminosity equal. Scrambled-face and scrambled-face-pareidolia images were created from the face images and face-pareidolia images, respectively. To determine whether the subjects experienced real faces or face pareidolia when they reported seeing a face, the scrambled images were used as control images. The scrambled versions of the real-face and face-pareidolia stimuli were created using the Shine Toolbox in Matlab.^11^ The scrambled conditions operated as controls to compare brain activity when scrambled stimuli were presented with activity when real-face and face-pareidolia (Fig.1) stimuli were shown.

### Procedure

Anatomical data collection was performed, with a focus on obtaining predominantly high-resolution sagittal T1 images using the following essential parameters: voxel size: 1 × 1 × 1 mm^3^; TR: 2600 ms; TE: 3.02 ms; FA: 80; FOV: 256 × 224 mm^2^; phase direction: anterior–posterior; and number of slices: 176. Finally, the real-face and face-pareidolia paradigms were presented to obtain functional data using a gradient echo sequence with predominantly T2 images with the following parameters: this sequence: voxel size: 3 × 3 × 3 mm^3^; TR = 2000 ms; TE = 40 ms; FA = 710; FOV = 192 × 192 mm^2^; phase direction: parallel to calcarine sulcus; and number of slices: 26.

The study paradigm comprised two parts. In the first part, real-face and scrambled real-face stimuli were shown in a block (Fig. 1- upper row), and in the second part, face-pareidolia and scrambled-face-pareidolia stimuli were shown in a block (Fig.1- lower row); each part included 10 blocks and the total stimulus consisted of 160 images. A red point on a black square was presented as a fixation point (12 s) between the face-scrambled and face pareidolia-scrambled stimuli to increase the focus of attention and eliminate eye movement. In each block, three images were selected randomly and shown for 3 s (Fig. 1), and the subjects were asked to push the button if they perceived any real face or face-like images among the three stimuli. Each part was completed within 42 seconds with the presentation of normal and scrambled versions and fixation points. A limitation of this study is that the data were obtained solely from the neural responses. We didn’t record a motor response.

### fMRI Data Analysis

Statistical Parametric Mapping eight software (SPM8; Wellcome Trust Centre for Neuroimaging, University College London) and FSL Software 5.0^12^ were used to analyse the raw fMRI data set, and SPM 8 was used to convert the raw 3D fMRI data to a 4D.nii format. In the first-level analysis, three levels of two conditions were examined for the real-face; face, scrambled, and face versus scrambled face (S > F) and face-pareidolia; pareidolia, scrambled, pareidolia versus scrambled pareidolia (S > P) conditions. The main step of first-level Feat analysis was Stats step which try to model the time series that’s going on in each voxel in the data. The essential part of Stats-step including GLM analysis also known as multiple regression which gave us comparison and contrast of two main conditions by telling which part of brain was activated in response to stimuli. We chose Full model set-up; Convolution was chosen as double-Gama HRF and Basic Shape was chosen as custom three column format. To find the significant percentage of activated areas in the brain, we used Harvard-Oxford Cortical and Subcortical Structural Atlas.

## RESULTS

**Fig.2:**
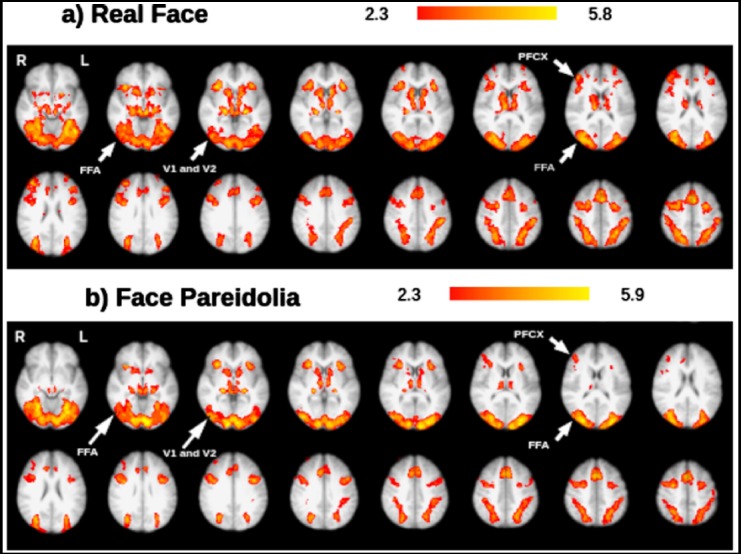
Representative set of brain images showing brain activation under the a) real-face condition and b) face-pareidolia condition; results are based on group analysis FFA: Fusiform face area, V1: Occipital cortex V1,V2: Occipital cortex V2, PFCX: Prefrontal cortex.

After completing group analyses of the 18 subjects under each condition (real face, face pareidolia, scrambled face, scrambled pareidolia, S > F, S > P), the activated areas were examined using FSL view; activation under the real-face and face-pareidolia conditions was of primary interest. Based on the real-face and face-pareidolia results, which were labelled by cope1feat analysis, the whole-brain analysis revealed activation in several specific brain areas including the prefrontal cortex (PFCX), occipital cortex V1, occipital cortex V2, and inferior temporal regions (such as the FFA; Fig.2). The major findings from the real face paradigm were specific activations in the right FFA (peaks at right FFA) and right PFCX (peaks at right PFCX (Fig.2a). There was also a smaller degree of activation in the right PFCX during the face-pareidolia paradigm (Fig.2b).

**Fig.3:**
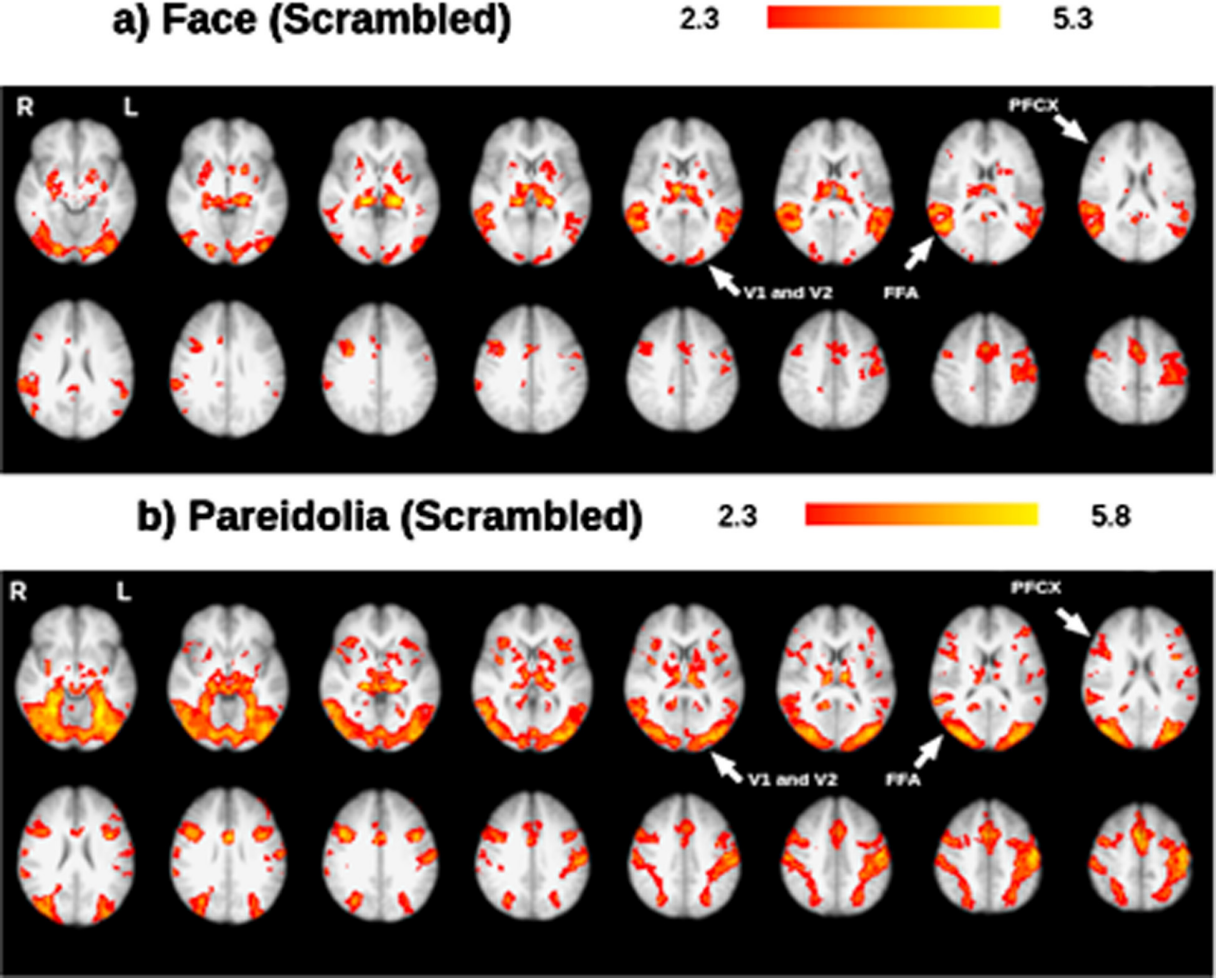
Representative set of brain images showing brain activation under the a) scrambled-face condition and b) scrambled-pareidolia condition; results are based on group analysis.

**Fig.4:**
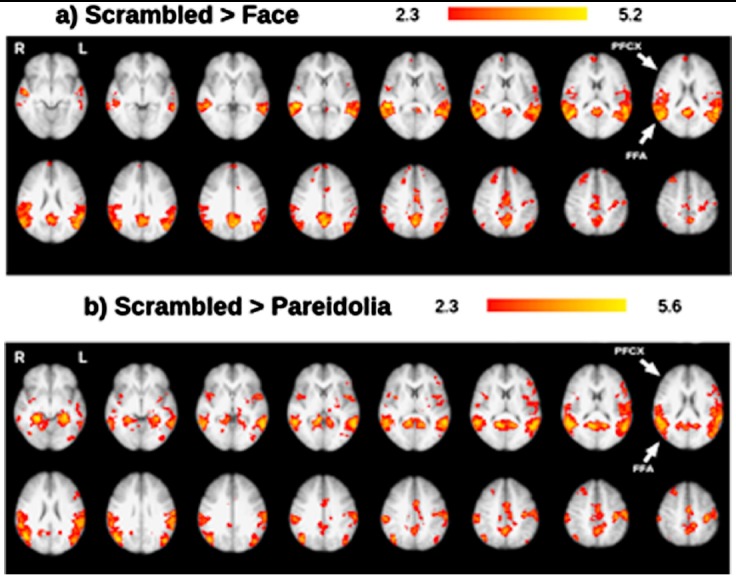
Representative set of brain images showing brain activation under the a) scrambled-face condition > face and b) scrambled-pareidolia > pareidolia condition; results are based on group analysis.

In both the scrambled-face and scrambled-pareidolia paradigms, brain activation was observed in the occipital cortex V1 (Fig. 3) and temporal cortex. Under the scrambled face condition, activation was observed in the FFA (Fig. 3a), whereas under the scrambled pareidolia condition, higher activation was observed in the occipital cortex V2 and occipital cortex V1. Additionally, specific activation was observed in the right PFCX under the scrambled pareidolia condition (Fig. 3b). Under both the scrambled-face and scrambled-pareidolia conditions, brain activation was observed in the occipital cortex V1, occipital cortex V2, temporal cortex, and PFCX. Under the scrambled face condition, the right PFCX showed minimal activation, and higher activation was observed in the right FFA than in the left FFA. Under the scrambled pareidolia condition, the FFA showed bilateral activation, and the right PFCX showed higher activation than did the left PFCX. The inferior temporal cortex was consistently more active during the real-face paradigm than during the scrambled-face paradigm (Fig. 4a), and activation was also greater under the pareidolia paradigm compared to the scrambled-pareidolia paradigm (Fig. 4b). Under both conditions, there was no activation in the PFCX (Fig. 4) when the real-face versus the scrambled-face and the pareidolia versus the scrambled-pareidolia comparisons were run.

## DISCUSSION

Comprehensive studies^13^-^20^ have investigated which brain regions participate in the processing of real-face and face-pareidolia stimuli. However, the brain regions that exhibit activation during these processes have yet to be fully determined. The present study found that certain brain regions are involved in processing real-face and face-pareidolia stimuli.

Despite their lack of experience, newborn infants preferentially look at faces over other objects, which suggests that the brain regions responsible for face recognition are functioning at birth.^21^-^23^ However, other studies have suggested that face-specific regions become functional following experience with visual stimuli during one’s lifetime.^24^-^26^ Nonetheless, faces remain the most important stimuli for visual perception studies. Furthermore, face pareidolia is more common than object pareidolia because the human visual system is highly sensitive to and adapted for the recognition of faces. Thus, for the first time, the present study employed a face-pareidolia paradigm to conduct a comprehensive investigation assessing the neurobiological mechanisms underlying the relationship between real-face and face-pareidolia perception using fMRI.

The whole-brain analysis revealed real-face- and face-pareidolia-specific activation in the FFA, PFCX, and sub-lobar regions such as the occipital cortex (V1 and V2). Face-pareidolia perception, which recruits both frontal and occipitotemporal regions, requires coordination of bottom-up and top-down regulation.^27^,^28^ In the present study, activation was observed in the PFCX and FFA, which might be responsible for the perception of illusory faces. These findings support those of Summerfield et al.,^8^ who reported activation in the FFA, medial-frontal lobe, and right parietal region when a house was misperceived as a face. Face expectations can enhance activation of the FFA.^29^ In the present study, bilateral FFA activation was observed under both the real-face and face-pareidolia conditions. The bilateral activation of the FFA during the face-pareidolia condition may have resulted from the expectation of seeing faces rather than from face-pareidolia mechanisms. It is possible that this bilateral activation may be enhanced by bottom-up processes, which could aid in understanding the neural mechanisms of real-face and face-pareidolia processing. Right PFCX activation was observed under both conditions, but left PFCX activation was found only under the real-face condition. These findings suggest that the left PFCX may play a lesser role during face-pareidolia processing. The FFA exhibited equal responses under the real-face and face-pareidolia conditions, which agrees with the results reported by Hadjikhani et al.,^30^ who found that the FFA showed equal responses to face-like objects and faces.

Fairhall and Ishai^31^ reported that lower visual cortices, such as the occipitotemporal cortex, may separate face-like properties from external information and then transport this information to a higher visual region, such as the FFA, for face perception. A recent Electroencephalograph (EEG) study^32^ recorded N170, a location in the bilateral occipitotemporal cortex that is sensitive to faces, and found that eyes were processed first, then other parts of the face were coded, and finally, face perception occurred after integration of this information. When participants were presented with real-face and face-pareidolia images in the present study, activation was observed in the occipitotemporal cortices (lower visual area), followed by the FFA, and then, one aspect of the PFCX (higher visual area) became active. However, it was not possible to determine whether the higher visual areas became active simultaneously or at different times.

Bilateral FFA activation was observed under both the face-pareidolia and scrambled-pareidolia conditions. The right PFCX showed activation under the face-pareidolia condition, and the right PFCX had higher activation than the left PFCX under the scrambled-pareidolia condition. Liu et al.^4^ suggested that the specific activation of the right FFA for face pareidolia could represent integration of bottom-up and the top-down signals. To extend this suggestion, it is possible that the integration between bottom-up and top-down signals may be driven by the right PFCX. This integration would ensure that the internal face template stored in the human brain and the sensory input match correctly.^33^

On the other hand, a comparison of brain activity under the S > F and S > P conditions did not reveal any activity in the PFCX for the S > P condition, but did reveal minimal activation for the S > F condition. PFCX activation was expected under both of these conditions but none was evident under the S > P condition. In contrast, the left FFA showed greater activation under the S > P condition than under the S > F condition. It is possible that if the sample size of this study were increased, the differences between the S > F and S > P conditions would be smaller, and a similar degree of FFA activation might have been observed under the S > P condition.

### Future Plans

Findings of the present study might be extended by collecting clinical data. For example, it would be possible to apply the same procedure to clinical patients with bipolar disorder or schizophrenia because these groups tend to experience hallucinations. Uchiyama et al.^34^ found that visual pareidolia may be related to visual illusions, and research on pareidolia might inform our understanding of visual hallucinations.
